# Behavioral sensitization induced by methamphetamine causes differential alterations in gene expression and histone acetylation of the prefrontal cortex in rats

**DOI:** 10.1186/s12868-021-00616-5

**Published:** 2021-04-06

**Authors:** Hui Li, Jing-An Chen, Qian-Zhi Ding, Guan-Yi Lu, Ning Wu, Rui-Bin Su, Fei Li, Jin Li

**Affiliations:** 1 State Key Laboratory of Toxicology and Medical Countermeasures, Beijing Key Laboratory of Neuropsychopharmacology, Beijing Institute of Pharmacology and Toxicology, Beijing, People’s Republic of China; 2 Beijing Institute of Radiation Medicine, Beijing, People’s Republic of China; 3 Beijing Institute of Pharmacology and Toxicology, 27th Taiping Road, 100850 Beijing, China

**Keywords:** Methamphetamine, Behavioral sensitization, Differentially expressed genes, Histone acetylation, ANP32A, POU3F2

## Abstract

**Background:**

Methamphetamine (METH) is one of the most widely abused illicit substances worldwide; unfortunately, its addiction mechanism remains unclear. Based on accumulating evidence, changes in gene expression and chromatin modifications might be related to the persistent effects of METH on the brain. In the present study, we took advantage of METH-induced behavioral sensitization as an animal model that reflects some aspects of drug addiction and examined the changes in gene expression and histone acetylation in the prefrontal cortex (PFC) of adult rats.

**Methods:**

We conducted mRNA microarray and chromatin immunoprecipitation (ChIP) coupled to DNA microarray (ChIP-chip) analyses to screen and identify changes in transcript levels and histone acetylation patterns. Functional enrichment analyses, including Gene Ontology (GO) and Kyoto Encyclopedia of Genes and Genomes (KEGG) analyses, were performed to analyze the differentially expressed genes. We then further identified alterations in ANP32A (acidic leucine-rich nuclear phosphoprotein-32A) and POU3F2 (POU domain, class 3, transcription factor 2) using qPCR and ChIP-PCR assays.

**Results:**

In the rat model of METH-induced behavioral sensitization, METH challenge caused 275 differentially expressed genes and a number of hyperacetylated genes (821 genes with H3 acetylation and 10 genes with H4 acetylation). Based on mRNA microarray and GO and KEGG enrichment analyses, 24 genes may be involved in METH-induced behavioral sensitization, and 7 genes were confirmed using qPCR. We further examined the alterations in the levels of the ANP32A and POU3F2 transcripts and histone acetylation at different periods of METH-induced behavioral sensitization. H4 hyperacetylation contributed to the increased levels of ANP32A mRNA and H3/H4 hyperacetylation contributed to the increased levels of POU3F2 mRNA induced by METH challenge-induced behavioral sensitization, but not by acute METH exposure.

**Conclusions:**

The present results revealed alterations in transcription and histone acetylation in the rat PFC by METH exposure and provided evidence that modifications of histone acetylation contributed to the alterations in gene expression caused by METH-induced behavioral sensitization.

## Background

Methamphetamine (METH) has been one of the most widely abused illicit substances worldwide for many years. Abusers exhibit deficits in cognition, behavior and emotions with long-term use and have a high METH relapse potential. The neurobiological mechanism of METH addiction is attributed to adaptations in signaling cascades and neural plasticity in the central nervous system. The enduring effects of these drugs, even after long periods of abstinence, have suggested the presence of persistent molecular events caused by transcriptional, epigenetic and translational changes [1] within addiction-related brain regions [2], such as the nucleus accumbens (NAc), prefrontal cortex (PFC), and hippocampus. Notably, accumulating evidence has revealed an important role of epigenetic regulation in the effects of psychostimulants [3] (e.g., cocaine [4, 5]) and identified the involvement of modifications of histones present in chromatin [6, 7], DNA methylation [8, 9], and DNA hydroxymethylation in the development of addiction. With respect to METH, however, only a few studies have investigated the epigenetic effects of these drugs, mainly focusing on the NAc and striatum [10–14].

In animals, repeated exposure to METH produces behavioral sensitization, which is manifested as a progressive increase in locomotor responses that is elicited by a challenge dose of the drug after long-term withdrawal. Behavioral sensitization has been suggested to be a useful animal model of drug abuse that may reflect the properties of craving [15]. Alterations to the associated brain regions, such the PFC, are suggested to mediate the etiology and maintenance of METH-induced behavioral sensitization [16]. Repeated exposure to drugs such as cocaine or METH was also reported to produce long-lasting changes in synaptic plasticity in the ventral tegmental area (VTA), NAc, and PFC [17–19]. A study in nonhuman primates has shown that changes in the PFC following METH administration may persist for as long as 3.5 years [20].

In the present study, we focused on whether the changes in gene expression occurring in response to METH-induced behavioral sensitization are related to histone acetylation in the PFC. Given the importance of the PFC in the development of METH abuse, we screened the alterations in gene expression and histone acetylation in the PFC following METH-induced behavioral sensitization and further investigated the contribution of histone acetylation to the expression of the candidate genes in the different phases of the behavior model. Alterations in histone acetylation might, in part, affect the METH-induced changes in gene expression in the PFC.

## Materials and methods

### Subjects

Adult male Sprague-Dawley rats (Grade SPF, Beijing Animal Center, Beijing, China) weighing 160–180 g were used in this study. Animals were maintained on a 12/12-hour light/dark cycle (lights on at 6:30 and off at 18:30), with an ambient temperature set at 20 °C to 22 °C. Animals were housed in 470 × 300 × 200-mm cages and provided access to rat maintenance food and water ad libitum. During housing, animals were monitored twice daily, and no abnormal events regarding the health status were detected. All experiments using animals were conducted in accordance with the NIH Guide for the Care and Use of Laboratory Animals (8th edition) and the guidelines of the National Research Council ([2006] 398, China). All sections of this report adhere to the ARRIVE Guidelines for reporting animal research. A completed ARRIVE guidelines checklist is included.

## Reagents

METH-HCl was provided by the Beijing Public Security Bureau Forensic Medical Examination Center (China). The drug was dissolved in saline (0.9% NaCl).

## Behavior experiment

The locomotor activity was measured in chambers (40 cm×40 cm×65 cm) using Digbehv spontaneous activity monitors [21] (Shanghai Jiliang Software Technology Co. Ltd., Shanghai, China). The total distance of horizontal locomotor activity was recorded with a video camera placed above the chamber and analyzed with the Digbehv software SuperState v3.0 system (AniLab Software & Instruments Co., Ningbo, China).

**Fig. 1:**
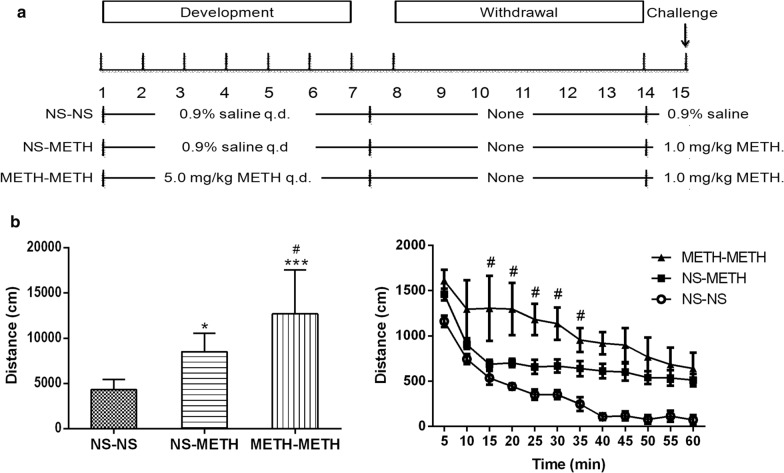
METH-induced behavioral sensitization. **a** Schematic of the behavioral sensitization training. **b** Total or time-dependent changes in the locomotion of rats with METH-induced behavioral sensitization within 60 min. Animals were injected once daily with METH (5 mg/kg, s.c.) followed by 7 days of withdrawal, and challenge was induced by a single METH (1.0 mg/kg, s.c.) injection. Data are presented as the means ± SEM of n = 8 rats per group. ***P < 0.001 and *P < 0.05 compared with the NS-NS group. ^#^*P* < 0.05 compared with the NS-METH group. One-way ANOVA followed by Tukey’s post hoc test. NS: 0.9% saline. METH: methamphetamine

The behavioral sensitization procedures were conducted using the method described in our previous study [14]. Behavioral sensitization consists of three phases (development, withdrawal and challenge phases) (Fig. 1a). The rats were administered METH (5 mg/kg, s.c.) or normal saline (NS) (1 ml/kg, s.c.) once daily in the acute phase or for 7 days in the development phase. On days 8–14, the rats were housed in home cages and did not receive injections in the withdrawal phase. On day 15, METH (1 mg/kg, s.c.) was administered to challenge the rats that were administered NS or METH in the development phase, and then the locomotor activity was recorded for 60 min in the challenge phase. The experiment was conducted using 40 rats (n =8 per group, 5 groups). A randomized METH and saline administration schedule was created using the standard = RAND() function in Microsoft Excel to reduce the number of animals used and the experimental error.

### RNA isolation and cDNA synthesis

Twenty-four hours after the behavioral experiment, rats were anesthetized with pentobarbital (70 mg/kg, i.p.) and then euthanized by decapitation. PFC tissues were dissected (+ 5.16 mm to 3.00 mm from the bregma) according to the stereotactic atlas published by Paxinos and Watson. Frozen PFC tissues were thawed in TRIzol (Invitrogen, Cat. No. 15,596,018) and then processed according to the manufacturer’s protocol. Total RNA was reverse-transcribed using the ReverTra Ace qPCR RT Kit (Qiagen, Cat. No. 205,313).

### Real‐time PCR

Real-time PCR was performed using a SYBR Green PCR kit (Qiagen, Cat. No. 204,145) and quantified using the 2^−ΔΔCt^ method. Real-time PCR was then performed using an ABI 7300 Real-Time PCR System (Applied Biosystems, Foster City, CA), amplified using SYBR Green (TOYOPO, Cat. No. QPS-201) and quantified using the 2^−ΔΔCt^ method. The primers for the analyzed genes are shown in Additional file 1: Table S1. Each sample was assayed in duplicate and normalized to the GAPDH levels.

### ChIP assay

PFC tissues were crosslinked with 1% formaldehyde at 37 °C for 10 min, and then glycine was added to a final concentration of 0.125 M and incubated for 5 min to stop the crosslinking. Samples were centrifuged at 800*g* at 4 °C for 10 min to pellet tissues. The PFC tissues were sonicated on wet ice in 300 µl of nuclear lysis buffer using a sonicator (Sonics Vibra-cell™, Sonics & Materials Inc., Newtown, Connecticut, USA). The sonication conditions were used: amplitude 70%, pulse 10 sec, and pause 59 sec for 9 cycles. The supernatant was collected as described in the instructions of a chromatin immunoprecipitation kit (Millipore, Cat. No. 17408). The lysate was precipitated with antibodies against acetyl-histone H3 (Millipore, Cat. No. 06599) and acetyl-histone H4 (Millipore, Cat. No. 06866). The following primer sequences were used for ChIP-PCR: POU3F2: forward primer 5′-CAGTACCGCTGAATAACGC-3′ and reverse primer 5′-CTGAGCCTGCTTACGATGA-3′; ANP32A: forward primer 5′-CCATCGGAGCATGTTTCGT-3′ and reverse primer 5′-AGGGTGAGATCGCCGTCTT-3′. The PFC samples used for qPCR and ChIP assay were obtained from the rats in another behavioral experiment, which were different from the samples used in microarray experiments. The samples were processed separately to perform the qPCR and ChIP experiments.

### Microarray experiments

Twenty-four hours after the behavioral experiment, the samples extracted from the PFCs of 8 rats per group were mixed and processed for the mRNA microarray or ChIP on chip DNA microarray. The mRNA microarray analysis was conducted using the Rat Whole Genome One Array that examines 19,803 unique genes using 24,358 probes (Phalanx Biotech Group, Hsinchu, Taiwan). The fluorescently amplified RNA targets were prepared from 2.5 µg of total RNA samples using the OneArray® Amino Allyl aRNA Amplification Kit (Phalanx Biotech Group), labeled with Cy5 dye (Amersham Pharmacia, Piscataway, NJ, USA), and then hybridized to the Rat Whole Genome OneArray® (ROA 1.1) (Phalanx Biotech Group) according to the Phalanx hybridization protocol. The slides were scanned using an Axon 4000 scanner (Molecular Devices, Sunnyvale, CA, USA). The Cy5 fluorescence intensity of each spot was analyzed using GenePix pro software (Molecular Devices). The signal intensity of each spot was loaded into the Rosetta Resolver System® (Rosetta Biosoftware) for data analysis.

For ChIP on chip, the DNA microarray analysis was performed using the Rat 3 × 720K RefSeq Promoter Array that examines 15,287 unique gene promoters overlapping 1,070 − 4,280 bp (Nimblegen). The input DNA was amplified using the WGA2 kit (Sigma, Cat. No. WGA2-50R) and purified with the QIAquick PCR purification kit (Qiagen). The product was labeled with Cy3 and Cy5 dyes and hybridized using a Roche NimbleGen hybridization kit. After drying, the slides were scanned with a Roche NimbleGen MS200, and the data were extracted using NimbleGen2.6 software. For each probe on the array, the log2 ratios of the Cy5-labeled ChIP DNA/Cy3-labeled input DNA were calculated using Signal MAP software. Peak detection was performed using NimbleScan software.

### Differential gene expression analysis and functional enrichment

Functional enrichment analyses, including Gene Ontology (GO) and Kyoto Encyclopedia of Genes and Genomes (KEGG) analyses, were performed to identify which differentially expressed genes were significantly enriched in GO terms and metabolic pathways at the thresholds of a corrected *P*-value [also named false discovery rate (FDR)] ≤ 0.05 and log2 fold change (log2 FC). GO functional enrichment and KEGG pathway analyses were conducted with the KOBAS program (http://kobas.cbi.pku.edu.cn/home.do) [22].

### Statistics

All data are presented as means ± SEM. The challenge data were assessed using one-way analysis of variance (ANOVA) followed by a post hoc test. For other data, a t-test was used. *P* values < 0.05 were considered statistically significant.

## Results

### METH-induced behavioral sensitization

The model of behavioral sensitization was established by 7 days of METH (5.0 mg/kg, s.c.) administration followed by 7 days of withdrawal and challenged with a single METH injection (1.0 mg/kg, s.c.) on day 15. One-way ANOVA showed a difference between the groups (*F*_(2, 21)_ = 14.58, *P* < 0.001). Rats in the METH sensitization group (METH-METH group) showed significantly greater locomotor activity than either the saline control (NS-NS) or METH acute treatment group (NS-METH) (Tukey’s post hoc test, *P* < 0.001 compared with the NS-NS group; *P* < 0.01 compared with the NS-METH group, Fig. 1b), indicating the increased locomotor response of sensitization. Compared with the NS-NS group, rats in the NS-METH group showed longer distances (*P* < 0.05, Fig. 1b).

### Changes in mRNA expression in the PFC of METH-induced behavioral sensitization rat

**Fig. 2:**
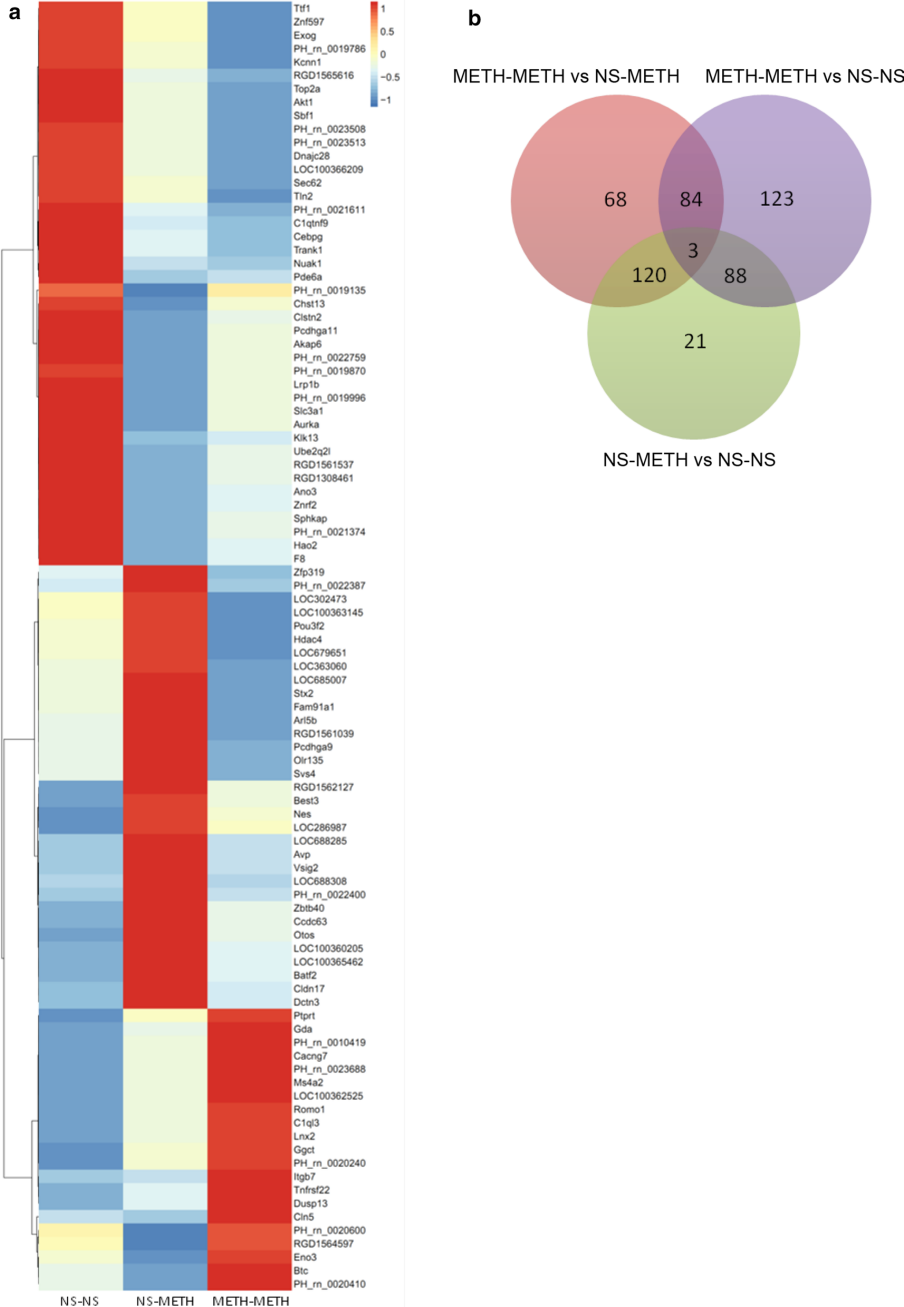
Heat map and Venn diagram of genes analyzed in PFC samples from rats subjected to METH-induced behavioral sensitization. **a** The heat map represents the expression levels of a panel of genes in different groups of rats experiencing METH-induced behavioral sensitization. **b** The Venn diagram depicts the overlap of genes identified in groups subjected to METH-induced behavioral sensitization

An mRNA microarray was performed to determine a genome-wide profile of METH-induced behavioral sensitization. The cut-off value was set at a fold change of ≤ 0.5 or ≥ 2.0 (*P* < 0.01). Figure 2 shows the clustering diagram and a heat map of the changes in mRNA expression in the NS-NS, NS-METH, and METH-METH groups. The expression of a large number of genes was altered by METH exposure (*P* < 0.01) (Fig. 2b). Among these, 275 genes were differentially expressed in the METH-METH group compared with the NS-METH group (97 genes were upregulated and 178 genes were downregulated), and 232 genes displayed differential expression in the NS-METH group compared with the NS-NS group (122 genes were upregulated and 110 genes were downregulated) (Additional file 2: Table S2).

### Ontology and pathway analyses of differentially expressed mRNAs in the PFC of METH-induced sensitization rats

**Fig. 3:**
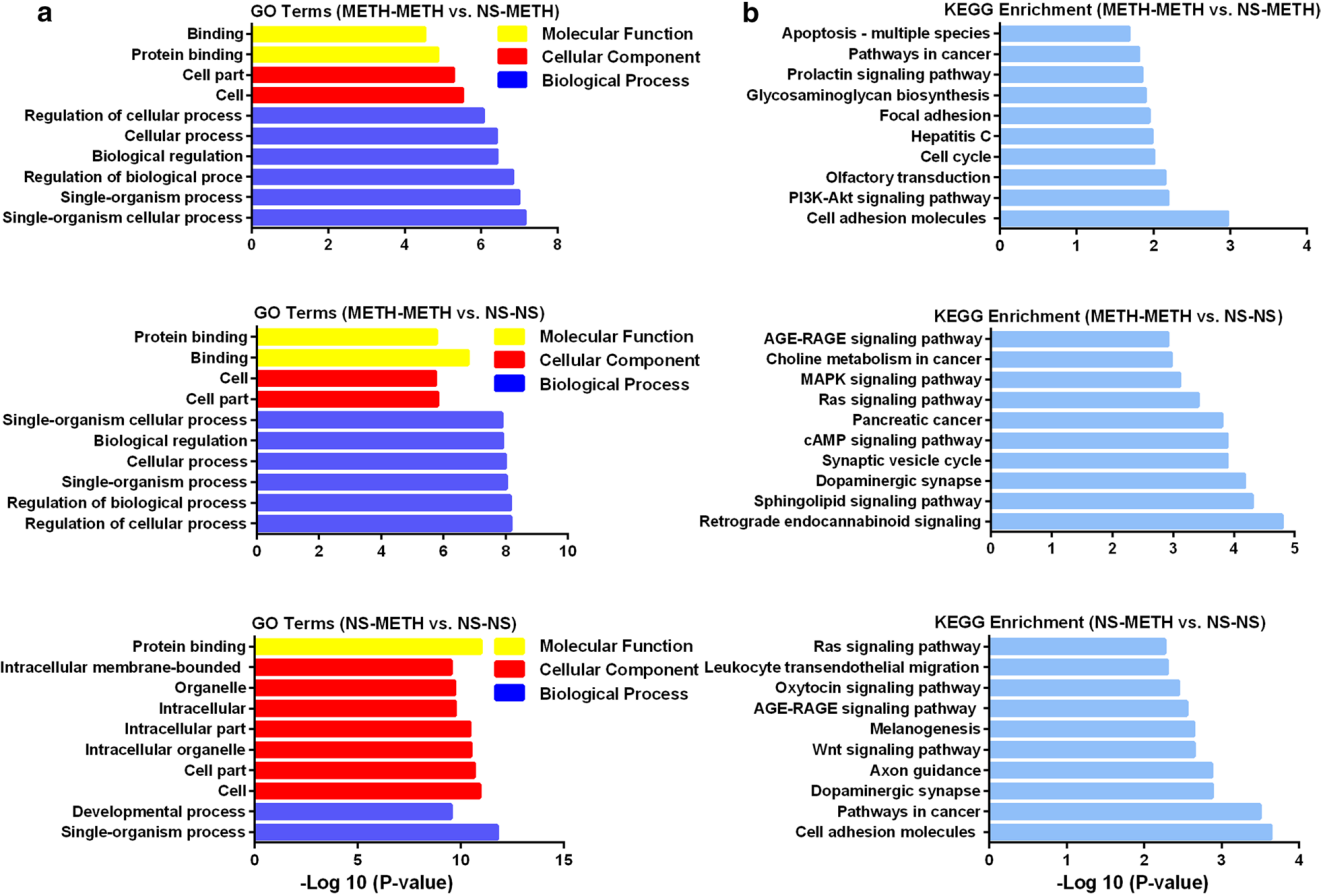
Functional enrichment of differentially expressed genes determined using GO (**a**) and KEGG (**b**) analyses. The horizontal axis represents the enrichment ratio

We performed a GO analysis and categorized the differentially expressed genes to investigate whether the clustering of differentially expressed genes induced by METH exposure correlates with functional categories. Figure 3a and Additional file 3: Table S3 show the top 10 ranked GO terms and the number of genes in each category. Compared with the acute METH treatment (NS-METH), the biological processes that were markedly increased in the METH-induced behavioral sensitization (METH-METH) group included biological regulation, cellular process and organism process. Regarding the molecular function, binding and protein binding were enriched in the METH-METH group.

We further performed a KEGG pathway enrichment analysis to assess the functional features of METH-induced behavioral sensitization-mediated alterations in gene sets. The top 10 significantly altered pathways are shown in Fig. 3b and Additional file 4: Table S4. Many signal transduction pathways were enriched in the METH-induced behavioral sensitization group, including cell adhesion, PI3K-AKT signaling pathway, olfactory transduction, and cell cycle (METH-METH group vs. NS-METH group, *P* < 0.0001). For the METH acute treatment (NS-METH) group, the genes categorized by the KEGG analysis were enriched in the cell adhesion, the pathway in cancer, and dopaminergic pathway.

### Changes in histone acetylation in the PFC of METH-induced sensitization rats

**Fig. 4:**
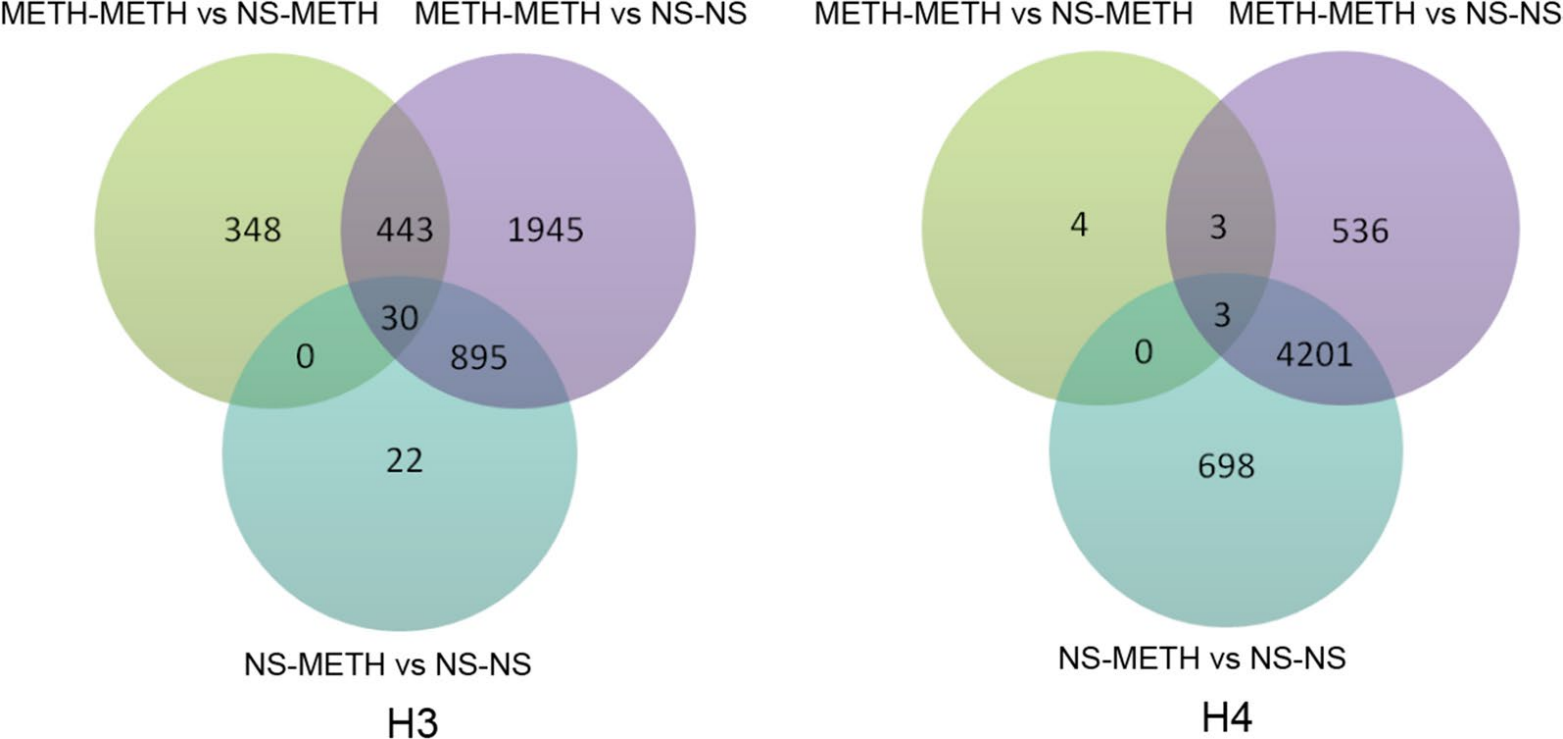
Venn diagram of genes analyzed in PFC samples from rats displaying METH-induced behavioral sensitization. The Venn diagrams show the overlapping genes labeled by each of the increased histone H3/H4 acetylation modifications following different METH administration paradigms

ChIP coupled with a DNA microarray analysis revealed that METH increased histone acetylation (H3 or H4) on a large number of gene promoters (Fig. 4), in accordance with mRNA activation. Compared with the NS-METH group, the METH-METH group induced many more acetylation modifications of H3 than H4 on the promoters; specifically, 821 genes presented H3 hyperacetylation and 10 genes showed H4 hyperacetylation (Additional file 5: Table S5). The NS-METH treatment also induced hyperacetylation on the gene promoters, including 947 genes with H3 hyperacetylation and 4902 genes with H4 hyperacetylation. Very little H3/H4 hyperacetylation overlapped between the METH-METH and NS-METH groups.

### Real‐time PCR confirmation of the candidate genes

**Fig. 5:**
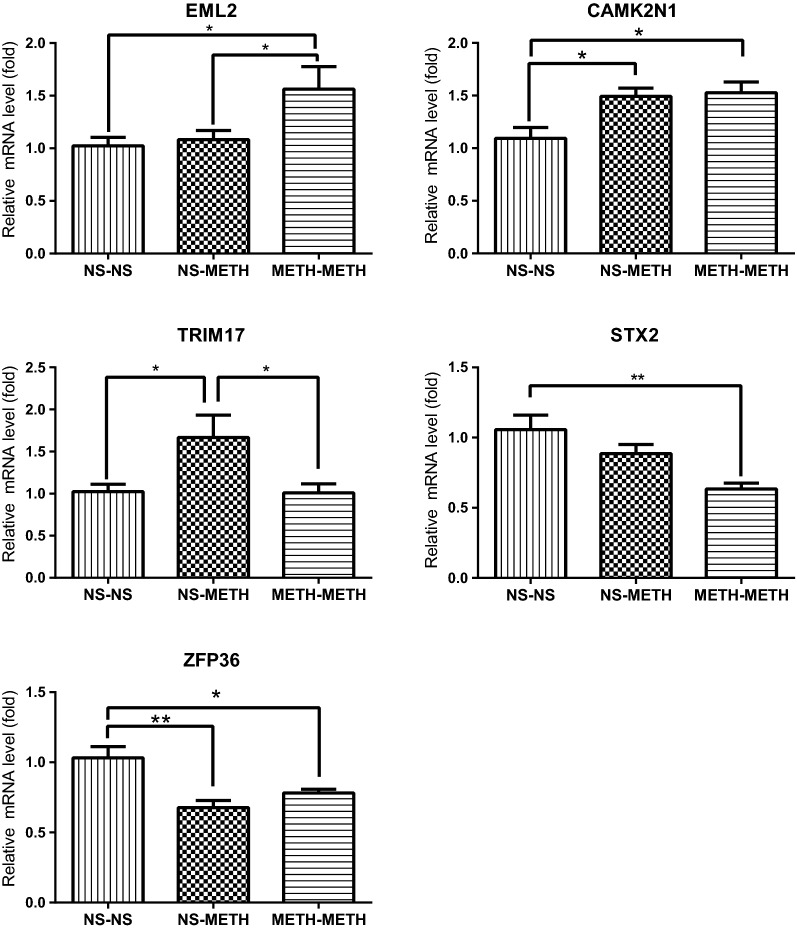
Confirmation of changes in gene expression using real-time PCR in the PFC samples from rats with METH-induced behavioral sensitization. Rats displaying METH-induced sensitization were challenged with a METH single injection. Data are presented as the means ± SEM, n = 6–9 rats per group. *P < 0.05 and **P < 0.01, one-way ANOVA followed by Tukey’s post hoc test

**Fig. 6:**
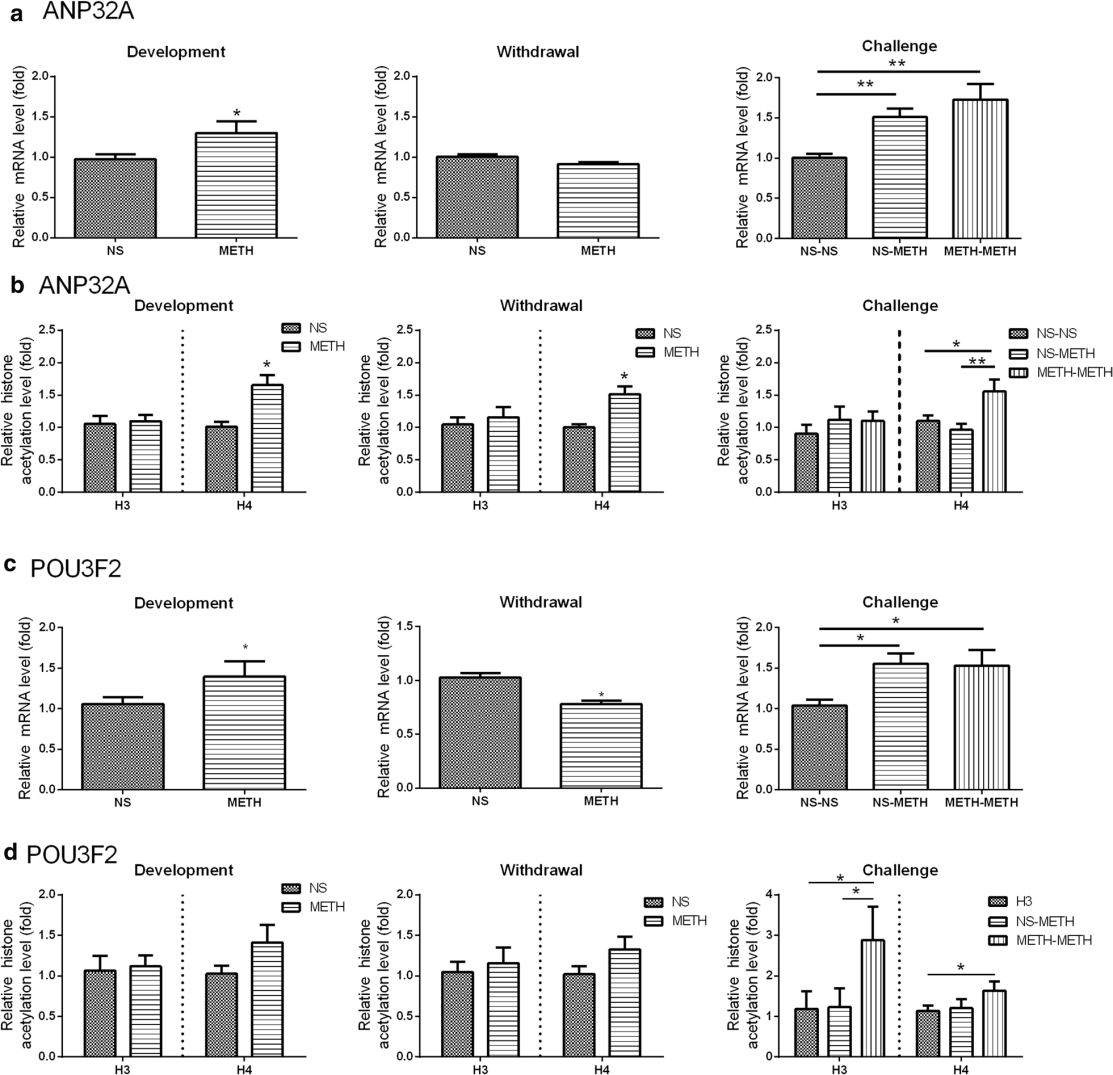
Validation of changes in the mRNA expression and H3/H4 histone acetylation of ANP32A (**a**, **b**) and POU3F2 (**c**, **d**). The results of real-time PCR and ChIP-PCR analyses revealed the changes in ANP32A and POU3F2 expression in the different phases of METH-induced behavioral sensitization. Data are presented as the means ± SEM, n = 9–10 rats per group for the ANP32A analysis and 14 rats per group for the POU3F2 analysis. *P < 0.05, **P < 0.01, one-way ANOVA followed by Tukey’s post hoc test for the challenge test and t-test for the development or withdrawal test

Based on the mRNA microarray data, GO/KEGG enrichment analyses, and the analysis of the functional properties, the genes listed in Additional file 6: Table S6 were likely to be associated with METH-induced behavioral sensitization. Of these genes, AVP, BCL2L1, EGR1, E2F3, LNX2, SHOC2, STX2 and ZFP36 clustered into the top 10 GO and KEGG categories, and ANP32A, EML2, EXOG, HIRA, METRN, POU3F2, STK32, SYT8, and TRIM17 were included in the GO category. According to the analysis of the GO categories, the expression of LNX2, SHOC2 and STX2 was altered by a single METH injection, and the expression of the other genes listed above was affected by METH-induced behavioral sensitization (METH-METH) but not the acute METH treatment (NS-METH). Furthermore, all of the genes displayed H3/H4 hyperacetylation. We then used qPCR to confirm the expression of some genes of interest. METH challenge in the behavioral sensitization model caused a significant increase in the mRNA levels of acidic nuclear phosphoprotein 32 family member A (ANP32A), calcium/calmodulin-dependent protein kinase II inhibitor 1 (CAMK2N1), echinoderm microtubule-associated protein-like 2 (EML2) and POU class 3 homeobox 2 (POU3F2), and a decrease in syntaxin 2 (STX2), tripartite motif-containing 17 (TRIM17), and zinc finger protein 36 (ZFP36) (*P* < 0.05, one-way ANOVA followed by Turkey’s post hoc test, Figs. 5 and 6).

### Alterations in ANP32A and POU3F2 expression in the development, withdrawal and challenge periods of METH-induced sensitization

Then, we selected ANP32A and POU3F2 and further measured the mRNA expression and histone modification of these two genes. In addition to the challenge of behavioral sensitization, the development and withdrawal phases were also involved to investigate the changes in ANP32A and POU3F2 expression throughout the whole process of METH-induced behavioral sensitization. After a chronic METH (5 mg/kg, s.c.) treatment for 7 days (development phase), the levels of the ANP32A mRNA and H4 acetylation were markedly increased compared with the NS group (*P* < 0.05, t-test, Fig. 6a, b). However, the expression of the ANP32A mRNA returned to the normal level, while H4 acetylation remained at a high level after 7 days of withdrawal (*P* < 0.05, t-test). Then, an injection of METH (1 mg/kg, s.c.) was administered as a challenge on day 15. One-way ANOVA showed a significant difference in mRNA expression (*F*_(2, 18)_ = 8.824, *P* < 0.01) and H4 acetylation (*F*_(2, 26)_ = 6.072, *P* < 0.01) between groups, and Tukey’s post hoc test showed that the challenge induced the expression of ANP32A mRNA (*P* < 0.01, METH-METH vs. NS-NS) and H4 acetylation (*P* < 0.01, METH-METH vs. NS-METH, *P* < 0.05, METH-METH vs. NS-NS) in the METH pretreatment group (METH-METH). The injection of METH alone also increased the level of the ANP32A mRNA (*P* < 0.05, NS-METH vs. NS-NS), but did not affect histone acetylation.

We next measured the change in levels of the POU3F2 mRNA and histone modification. The expression of the POU3F2 mRNA was significantly increased by the chronic METH treatment for 7 days and decreased after 7 days of withdrawal (*P* < 0.05, t-test), while H3 and H4 acetylation were not affected by the treatment (Fig. 6c, d). In the challenge phase, one-way ANOVA showed that challenge with a low dose of METH caused a significant difference in mRNA expression (*F*_(2, 45)_ = 4.01, *P* < 0.05) and H3 and H4 hyperacetylation (H3: *F*_(2, 36)_ = 4.17, *P* < 0.05; H4: *F*_(2, 38)_ = 3.47, *P* < 0.05) between groups. Tukey’s post hoc test revealed a significant increase in POU3F2 mRNA expression (*P* < 0.05, METH-METH vs. NS-NS) and H3 and H4 hyperacetylation in the behavioral sensitization group (H3: *P* < 0.05, METH-METH vs. NS-NS, METH-METH vs. NS-METH; H4: *P* < 0.05, METH-METH vs. NS-NS). Notably, the single METH injection (NS-METH) also increased the expression of the POU3F2 mRNA (*P* < 0.05, vs. NS-NS), but not the levels of H3/H4 acetylation.

## Discussion

Behavioral sensitization is an animal model that reflects some aspects of drug addiction, such as drug seeking and relapse behaviors, but the neurobiological mechanism has not yet been completely elucidated. Drug-induced changes in gene expression and sustained changes in genes regulated by epigenetic mechanisms in addiction-associated regions are thought to contribute to long-lasting behavioral abnormalities and neuroadaptation. The present study examined the transcriptional changes in gene expression and alterations in histone acetylation in the PFC of adult rats that experienced the different phases of METH-induced behavioral sensitization to further analyze the neurological mechanism underlying the development of METH addiction. The mRNA microarray and ChIP microarray analyses revealed 275 differentially expressed genes and significant hyperacetylation, including 821 genes with H3 hyperacetylation and 10 genes with H4 hyperacetylation, caused by METH-induced behavioral sensitization in the PFC, suggesting that histone hyperacetylation is associated with the increased expression of a set of genes caused by METH-induced behavioral sensitization.

Based on accumulating evidence, drug exposure causes modifications of global histone acetylation or specific changes in the gene-promoter regions in a complex manner. As shown in the studies by Wang et al. chronic self- or experimenter-administered cocaine induced long-term increases in global H3 and H4 acetylation in the NAc shell [23], and cocaine abstinence caused the acetylation of histone H3K9 and histone H4K8 in the PFC [24]. ChIP sequencing provides access to precise patterns of histone acetylation. According to Renthal et al. [25], chronic cocaine exposure produces a larger number of genes with H3 acetylation than H4 acetylation in the NAc. A set of genes, such as CBP in the NAc shell [26] or BDNF in the PFC [27], is activated by cocaine, and these changes are associated with the hyperacetylation of H3 and/or H4. Regarding METH exposure, Tracey et al.. reported time-dependent increases in the levels of acetylated H4K5 and H4K8 and decreases in the levels of acetylated H3K9, H3K18, and H4K16 in the NAc following a single METH injection [13]. González et al.. observed increased levels of total H3 acetylation and decreased H4 acetylation after a single METH injection, while repeated METH injections decreased levels of total H3 and H4 acetylation in the mPFC [28, 29]. As shown in the present study, METH-induced behavioral sensitization caused a large number of genes to exhibit H3 hyperacetylation and few genes exhibited H4 hyperacetylation, while more H4 hyperacetylation than H3 hyperacetylation was induced by acute METH exposure in the PFC.

METH-induced behavioral sensitization led to a change in the transcriptional profile of the PFC in the present study, as manifested by the large number of differentially expressed genes categorized by GO and KEGG pathway enrichment, including cell adhesion, the PI3K-AKT signaling pathway, olfactory transduction, and the cell cycle, compared with the acute METH treatment control. In fact, exposure to drugs, such as cocaine and morphine, has consistently been shown to induce cell adhesion [30] and activate intracellular signaling pathways, such as PI3K-AKT [31] and the prolactin signaling pathway [32], to mediate the effects of addiction. Some potential effects of the genes identified and confirmed in this study using qPCR on behavior have been identified in the previous reports. The expression of some genes encoding proteins involved in behavioral sensitization decreased (e.g., STX2, ZFP36 and TRIM17) and the expression of some genes increased (e.g., ANP32A, CAMK2N1, EML2 and POU3F2) at the mRNA level induced by METH challenge. These genes encode not only transcription factors but also proteins involved in neuron differentiation, synaptic plasticity and intracellular signaling. STX2 was reported to play a role in CNS damage [33–35]. STX2 interacts with neurons and promotes structural changes in the cell membrane that may indicate the synaptic vesicle cycle [36]. STX2 induces the PI3K-involved signaling pathway to release glutamate, which contributes to the CNS impairment [37]. EML2 encodes echinoderm microtubule-associated protein-like 2 protein, which plays a role in the formation of the mitotic spindle and interphase microtubule network [38]. The EML2 methylation status differs among individual cell types in the brain tissue [39], suggesting that EML2 is an important determinant of cell type-specific differentiation in the brain [40].

We further identified the changes in ANP32A and POU3F2 by conducting qPCR and H3/H4 acetylation measurements. ANP32A is mostly localized in the nucleus but is also present on the endoplasmic reticulum and in the cytoplasm [41]. To date, ANP32A has been reported to play roles in a variety of cellular functions, including RNA transport, transcription, cell-mediated cytotoxicity, and tumor suppression [42–44]. In the CNS, ANP32A contributes to the survival and differentiation of neurons [45–47], as well as synaptic plasticity and memory in aged mice or tau transgenic mice used as a model of Alzheimer’s disease. In the present study, levels of the ANP32A mRNA were increased by acute or chronic treatment with METH, as well as METH challenge-induced behavioral sensitization. The ChIP assay showed a robust induction of the acetylation of H4 at the promoter of ANP32A caused by chronic METH use or challenge-induced behavioral sensitization, but not by acute METH treatment. The lack of change in H4 hyperacetylation in response to acute METH exposure but its potential increase in the development and challenge stages of METH-induced behavioral sensitization suggested that H4 hyperacetylation at the ANP32A gene may have an important role in modulating the motivation for METH reinforcement.

POU3F2 (also known as BRN2), a class III POU protein [48], is prominently expressed in the neocortex and upregulated in the progenitor cells of the subventricular zone, intermediate zone and outer layer of the neocortex during the early stages of embryonic brain development [49, 50]. POU3F2 contributes to neural formation and cell fate determination and regulates cortical neural migration, neurogenesis and the positioning of cortical neurons [49, 51]. Furthermore, POU3F2 has been suggested to regulate tyrosine hydroxylase and tryptophan hydroxylase 2, the rate-limiting enzymes involved in the synthesis of dopamine and 5-HT [52], both of which are important for the regulation of psychological and physiological functions. POU3F2^Δ/Δ^ mice in which all three homopolymeric amino acid repeats were deleted from the POU3F2 transactivation domain displayed cognitive impairments in object recognition and object location tests [53], suggesting that POU3F2 is involved in cognitive function. In the present study, the expression of the POU3F2 mRNA was upregulated by METH exposure, including acute or chronic METH treatment, and METH challenge-induced behavioral sensitization. However, when METH was withdrawn after chronic treatment, the POU3F2 mRNA was downregulated instead. Notably, histone acetylation at the promoter of POU3F2 showed a specific change during the course of METH treatment, which manifested as both H4 and H3 hyperacetylation only in response to METH-induced behavioral sensitization but not acute or chronic METH treatment or withdrawal. The change in the patterns suggests that histone acetylation of POU3F2 is involved in the mechanism of neuroplasticity underlying the transition from recreational METH use to compulsive use. Moreover, POU3F2 is involved in schizophrenia [54, 55], the syndrome and pathology of which are very similar to METH addiction. Therefore, we speculated that POU3F2 potentially represents one of the common genetic bases underlying schizophrenia and METH use.

## Conclusions

We identified the changes in gene expression and the diverse alterations in histone acetylation in the PFC in the different phases of METH-induced behavioral sensitization. By further analyzing the levels of acetylated histones bound to the promoters of some genes (e.g., ANP32A and POU3F2), we revealed that histone modifications might participate in the transcriptional changes in gene expression caused by the specific manners of METH treatment, which further confirmed the epigenetic mechanisms of gene regulation underlying the long-lasting changes induced by drugs. Meanwhile, METH-induced histone acetylation might be necessary, but not sufficient, to induce changes in gene expression. In the present study, the functions of the genes of interest in the brain were not explored. The transcript levels and histone acetylation of the genes associated with functions in specific brain regions may provide new knowledge that will enable researchers to elucidate the neurobiological mechanism underlying METH addiction and develop alternative pharmacological methods for treating psychiatric disorders, which should be extensively studied in the near future.

## Supplementary Information

**Additional file 1: Table S1.** The primers for the genes in Real-time PCR.

**Additional file 2: Table S2.** Raw data of mRNA microarray in METH-induced behavioral sensitization.

**Additional file 3: Table S3.** The detailed information of top 10 GO terms and the categorized differential expression genes in the group of METH-METH vs. NS-METH, METH-METH vs. NS-NS, and NS-METH vs. NS-NS.

**Additional file 4: Table S4.** The detailed information of top 10 KEGG pathway enrichment and the categorized differential expression genes in the group of METH-METH vs. NS-METH, METH-METH vs. NS-NS, and NS-METH vs. NS-NS.

**Additional file 5: Table S5.** Raw data of ChIP-All peaks in METH-induced behavioral sensitization.

**Additional file 6: Table S6.** The information of genes of interest. The genes were selected based on the analysis of mRNA microarray, GO and KEGG enrichment.

## Data Availability

The datasets supporting the conclusions of this article are included within the article and its additional files. The sequencing data are available from Gene Expression Omnibus (GEO) database (https://www.ncbi.nlm.nih.gov/geo/query/acc.cgi?acc=GSE166096).

## Abbreviations

METH: Methamphetamine
ChIP: Chromatin immunoprecipitation
NAc: Nucleus accumbens
PFC: Prefrontal cortex
NS: Normal saline
GO: Gene ontology
KEGG: Kyoto Encyclopedia of Genes and Genomes
ANP32A: Acidic leucine-rich nuclear phosphoprotein-32A
POU3F2: POU domain, class 3, transcription factor 2
