# Supplementary material for: Behavioral sensitization induced by methamphetamine causes differential alterations in gene expression and histone acetylation of the prefrontal cortex in rats
Source: BMC Neurosci. 2021 Apr 6;22:24. doi: 10.1186/s12868-021-00616-5 (PMC8022387; doi:10.1186/s12868-021-00616-5)
Supplement: Supplementary file 1 — Additional file 1: Table S1. The primers for the genes in Real-time PCR. [file 12868_2021_616_MOESM1_ESM.docx]

The primers for the genes in Real-time PCR

| Anp32a | Sense: TCACCTCCATTTCCAACTTACC  Antisense: CATTTCTCTGCCAATACTTCCA |
| --- | --- |
| Camk2n1 | Sense: GCGTTGTTATTGAAGATGATAGGA  Antisense: ATTGTCTTTGGGGGCAGTTAG |
| Eml2 | Sense: GAGGAGCAGAGACAACGACAC  Antisense: AAGGTGGAAAGGGAAACTGAG |
| Pou3f2 | Sense: TTTCCTCAAATGCCCTAAGC  Antisense: CAAACTCTCACCACCTCCTTCT |
| Stx2 | Sense: GACTCAGGGTGAAATGGTCAAC  Antisense: GCTCTGGTATTTGATGGCTTTC |
| Trim17 | Sense: GCAAGAAAGGGAAGAAGAAGC  Antisense: TGGTGTATTTGGCACAGGTCT |
| Zfp36 | Sense: CGGAGGACTTTGGAACATAAAC  Antisense: CGAAGTAGGTGAGGGTGACAG |
| GAPDH | Sense: GTATTGGGCGCCTGGTCACC  Antisense: CGCTCCTGGAAGATGGTGATGG |
