# Supplementary material for: Behavioral sensitization induced by methamphetamine causes differential alterations in gene expression and histone acetylation of the prefrontal cortex in rats
Source: BMC Neurosci. 2021 Apr 6;22:24. doi: 10.1186/s12868-021-00616-5 (PMC8022387; doi:10.1186/s12868-021-00616-5)
Supplement: Supplementary file 6 — Additional file 6: Table S6. The information of genes of interest. The genes were selected based on the analysis of mRNA microarray, GO and KEGG enrichment. [file 12868_2021_616_MOESM6_ESM.pdf]

The information of genes of interest

Name	Entrez_gene	KEGG pathway or the biological process involved in neuropsychological diseases
ANP32A	25379	Inhibition of acetyltransferases, cell proliferation, differentiation and apoptosis
ATP5I	140608	ATP synthase, H+ transporting, mitochondrial F0 complex
AVP	24221	Neurophysin 2 specifically binds vasopressin
BCL2L1	24888	Neuronal apoptosis
CADM3	360882	Involved in the cell- cell adhesion, neuronal synapse formation.
CAMK2N1	287005	Specific inhibitor of CaM- kinase II
COX6A1	25282	Mitochondrial respiratory complex IV
COX8A	171335	Cytochrome c oxidase enzyme, mitochondrial respiratory chain
E2F3	291105	Control of cell- cycle progression, implication of cocaine responses , cell cycle
EGR1	24330	Regulation of cell survival, proliferation and cell death; responses to drug
EML2	192360	Microtubule nucleation and growth
EXOG	301062	Repression of histone gene transcription
HIRA	363849	Regulation of actin filament dynamics, downstream of several Rho family GTPase
LIMK1	65172	Neuronal proliferation and differentiation
LNX2	360761	Gial cell differentiation and axonal network formation
METRN	287151	Microtubule nucleation and growth
POU3F2	29588	Neural formation, migration, neurogenesis and positioning of neurons
SHOC2	309548	Ras signaling pathway
STK32C	365381	Protein serine/threoine kinase activity
STX2	25130	Epithelial morphogenesis, Synaptic vesicle cycle
SYT8	60566	Trafficking and exocytosis of secretory vesicles
TRIM17	64702	Function as an ubiquitin E3 ligase
USP9X	363445	Regulation of axonal growth and neuronal cell migration; Deubiquitinase
ZFP36	79426	Transcriptional activator
